# Sensitivity of an international notification system for wildlife diseases: A case study using the OIE‐WAHIS data on tularemia

**DOI:** 10.1111/zph.12916

**Published:** 2022-01-29

**Authors:** Angela Fanelli, Lina Awada, Paula Caceres‐Soto, François Diaz, Tiggy Grillo, Itlala Gizo, Keith Hamilton, Christine Leon Rolez, Peter Melens, Roberta Morales, Lina Mur, Sophie Muset, Lorenz Nake, Lesa Thompson, Chadia Wannous, Paolo Tizzani

**Affiliations:** ^1^ Department of Veterinary Medicine University of Bari Bari Italy; ^2^ World Animal Health Information and Analysis Department World Organisation for Animal Health (OIE) Paris France; ^3^ Preparedness and Resilience Department World Organisation for Animal Health (OIE) Paris France; ^4^ Engagement and Investment Department World Organisation for Animal Health (OIE) Paris France; ^5^ Regional Representation for Asia and the Pacific World Organisation for Animal Health (OIE) Tokyo Japan; ^6^ One Health World Organization for Animal Health (OIE) Regional Office for Africa Nairobi Kenya

**Keywords:** capture–recapture, notification system, OIE‐WAHIS, tularemia, veterinary epidemiology, wildlife disease

## Abstract

The World Organization for Animal Health (OIE) has recently developed a Wildlife Health Framework to respond to the need of members to manage the risk from emerging diseases at the animal–human–ecosystem interface. One of its objectives is to improve surveillance systems, early detection and notification of wildlife diseases. Members share information on disease occurrence by reporting through the OIE World Animal Health Information System (OIE‐WAHIS—formerly known as ‘WAHIS’). To evaluate the capacity of a surveillance system to detect disease events, it is important to quantify the gap between all known events and those officially notified to the OIE. This study used capture–recapture analysis to estimate the sensitivity of the OIE‐WAHIS system for a OIE‐listed wildlife disease by comparing information from publicly available sources to identify undetected events. This article presents a case study of the occurrence of tularemia in lagomorphs among selected North American and European countries during the period 2014–2019. First, an analysis using three data sources (OIE‐WAHIS, ProMED, WHO‐EIOS [Epidemic Intelligence from Open Sources]) was conducted. Subsequent analysis then explored the model integrating information from a fourth source (scientific literature collected in PubMed). Two models were built to evaluate both the sensitivity of the OIE‐WAHIS using media reports (ProMED and WHO‐EIOS), which is likely to represent current closer to real‐time events, and published scientific data, which is more useful for retrospective analysis. Using the three‐source approach, the predicted number of tularemia events was 93 (95% CI: 75–114), with an OIE‐WAHIS sensitivity of 90%. In the four‐source approach, the number of predicted events increased to 120 (95% CI: 99–143), dropping the sensitivity of the OIE‐WAHIS to 70%. The results indicate a good sensitivity of the OIE‐WAHIS system using the three‐source approach, but lower sensitivity when including information from the scientific literature. Further analysis should be undertaken to identify diseases and regions for which international reporting presents a low sensitivity. This will enable evaluation and prioritization of underreported OIE‐listed wildlife diseases and identify areas of focus as part of the Wildlife Health Framework. This study also highlights the need for stronger collaborations between academia and National Veterinary Services to enhance surveillance systems for notifiable diseases.

**Impacts**

- This is the first application of capture–recapture methodology to assess the sensitivity of a notification system for wildlife diseases.
- The OIE‐WAHIS system performed efficiently for tularemia when considering information coming from media reports, but it missed some events reported in the scientific literature.
- Assessment of the OIE‐WAHIS sensitivity provides valuable information on the surveillance system in place and the reporting behaviour of members.

## 1 INTRODUCTION

Wildlife can be both a target of and reservoir for pathogens capable of infecting domestic animals and humans. A majority of emerging infectious diseases are zoonoses with reservoirs in wildlife (Haider et al., 2020; Jones et al., 2008). Diseases  shared  at the animal–human–ecosystem interface can have a significant impact on public health, global economies, livelihood and biodiversity (Gortazar et al., 2015). The ongoing COVID‐19 pandemic represents one of the many examples of the potentially significant threats resulting from a spillover event from animals to humans when factors align (Plowright et al., 2017). Thus, surveillance of disease in wildlife is an essential tool for protecting both human and animal health through faster detection of threats and quicker responses.

Through its mandate, the World Organization for Animal Health (OIE) is actively involved in the reduction of disease spread, including surveillance and protection of wildlife. The OIE gathers worldwide information on wildlife diseases to provide Members with a good understanding of the epidemiological situation of selected diseases (OIE, 2021b). National Veterinary Services submit this information to the OIE, which verifies it and makes the data publicly available through the OIE World Animal Health Information System (OIE‐WAHIS—formerly known as ‘WAHIS’).

The OIE has recently established a Wildlife Health Framework with two main objectives: (a) improving the management of the risk of pathogen emergence in wildlife and transmission at the human–animal–ecosystem interface, and (b) improving surveillance systems, early detection, notification and management of wildlife diseases (OIE, 2021c). To achieve these objectives, specific activities have been planned, including undertaking comparisons between wildlife disease data collected by the OIE with that of other sources to identify reporting gaps and improve the sensitivity of the OIE‐WAHIS system. Assessment of the OIE‐WAHIS sensitivity provides valuable information on the surveillance system in place and the reporting behaviour of Members. Knowing limitations in countries' surveillance and reporting will help define actions to optimize the system and best allocate efforts and funds.

In this study, we used the capture–recapture (CR) method to estimate the OIE‐WAHIS sensitivity. CR sampling has been widely used to adjust for undercounting animal populations in the biological sciences (Schwarz & Seber, 1999; Seber, 1982, 1986, 1992). The CR technique developed from the need for accurate estimates of animal population sizes when individual observation of each animal is not feasible. Recapture information, also referred to as source‐overlap information or source intersection, can be used to estimate the number of missing records and thus the actual number of the population under monitoring. The same concept has been applied in epidemiology to obtain the real prevalence rates for various diseases and to evaluate the completeness of different sources for disease monitoring (Hook & Regal, 1995). We propose the application of a CR multi‐source approach to estimate the completeness of the OIE‐WAHIS wildlife diseases notification system. A case study on notifications of tularemia is used to illustrate the potential use of the method. Tularemia is a zoonosis caused by *Francisella tularensis*, which infects lagomorphs and other species, representing a public health threat. Tularemia is an OIE‐listed disease that occurs endemically in the Northern Hemisphere, and as epizootic outbreaks in countries in North America and Europe, while it occurs as sporadic cases in some other countries in Europe and Asia. It is rarely reported from the tropics or the Southern Hemisphere. Tularemia was considered a good test case for several reasons. Firstly, it has a limited range of susceptible hosts and clear clinical signs which enables even countries with limited diagnostic capabilities to detect the disease. Secondly, it is a zoonosis with an impact on public health, which usually means a better surveillance system is in place.

In the first instance, an analysis using three different data sources is presented. The three sources selected were all surveillance‐based, widely recognized and used, and with a special emphasis on early warning and rapid reporting for timely response. Afterwards, a second model was built integrating scientific literature as a fourth source of information. These different approaches were carried out to evaluate the usefulness of different sources in improving a system sensitivity. The use of media reports (retrieved with surveillance‐based sources) is more consistent with the purposes of an international surveillance system that needs to detect events in an almost real‐time way (rapid detection to serve a rapid reaction), while scientific literature is more useful for retrospective evaluations that are sometimes beyond the purposes of a surveillance system.

## 2 MATERIALS AND METHODS

### 2.1 Data sources

Data on tularemia events were extracted with no language restrictions from the following sources.

#### 2.1.1 OIE‐WAHIS system^1^

Tularemia is an OIE‐listed disease, and as such, OIE Members have a legal obligation to report information on occurrence of the disease to the OIE. The OIE‐WAHIS is a dynamic database, gathering information notified by the National Veterinary Services. Data reported in the OIE‐WAHIS can derive from two different reporting channels: (a) ‘Immediate notification reports’ when the occurrence of the disease in the country is considered an exceptional event and is reported within 24 hr after the confirmation of the event; or (b) ‘Six‐monthly reports’ if the disease has a more stable presence (considered endemic) in the country and is reported through semestrial updates. For most countries included in our study, tularemia is reported through the second type of report. The system is constantly updated, collecting ‘close to real‐time’ events; data included in this study refer to the information available as of 21 March 2021.

#### 2.1.2 Epidemic Intelligence from Open Sources (EIOS)^2^

Epidemic Intelligence from Open Sources (EIOS) is the epidemic intelligence system led by the World Health Organization (WHO) and used for scanning information from open sources to detect disease events. It was created to support public health and animal health experts with the early detection of One Health hazards. The system currently covers several infectious diseases in animals and humans, including tularemia. The system collects information from more than 15,000 sources and is constantly refreshed to gather ‘close to real‐time’ events.

#### 2.1.3 Program for Monitoring Emerging Diseases (ProMED)^3^

ProMED‐mail provides reports on outbreaks of diseases of infectious or toxic aetiology, affecting humans, animals and plants. Records on tularemia events were searched in the archives housed on the ProMED‐mail website. In this case, the system also collects ‘close to real‐time’ events.

#### 2.1.4 PubMed^4^

PubMed is a web interface for searching MEDLINE, a bibliographic database containing references of scientific journal articles. Information on tularemia events was derived by developing a comprehensive search strategy implemented in the PubMed Advanced Search Builder (Table S1). For the purpose of this study, we considered all publications reporting data collected during the study period, including papers which may have been published after 2019 but including disease events from the reporting period.

Dependence between the sources, defined as the existence of a direct causal effect of inclusion in a sample if a case is also included in another sample (Braeye et al., 2016), is presented in the Table S2.

### 2.2 Event definition and study area

**FIGURE 1:**
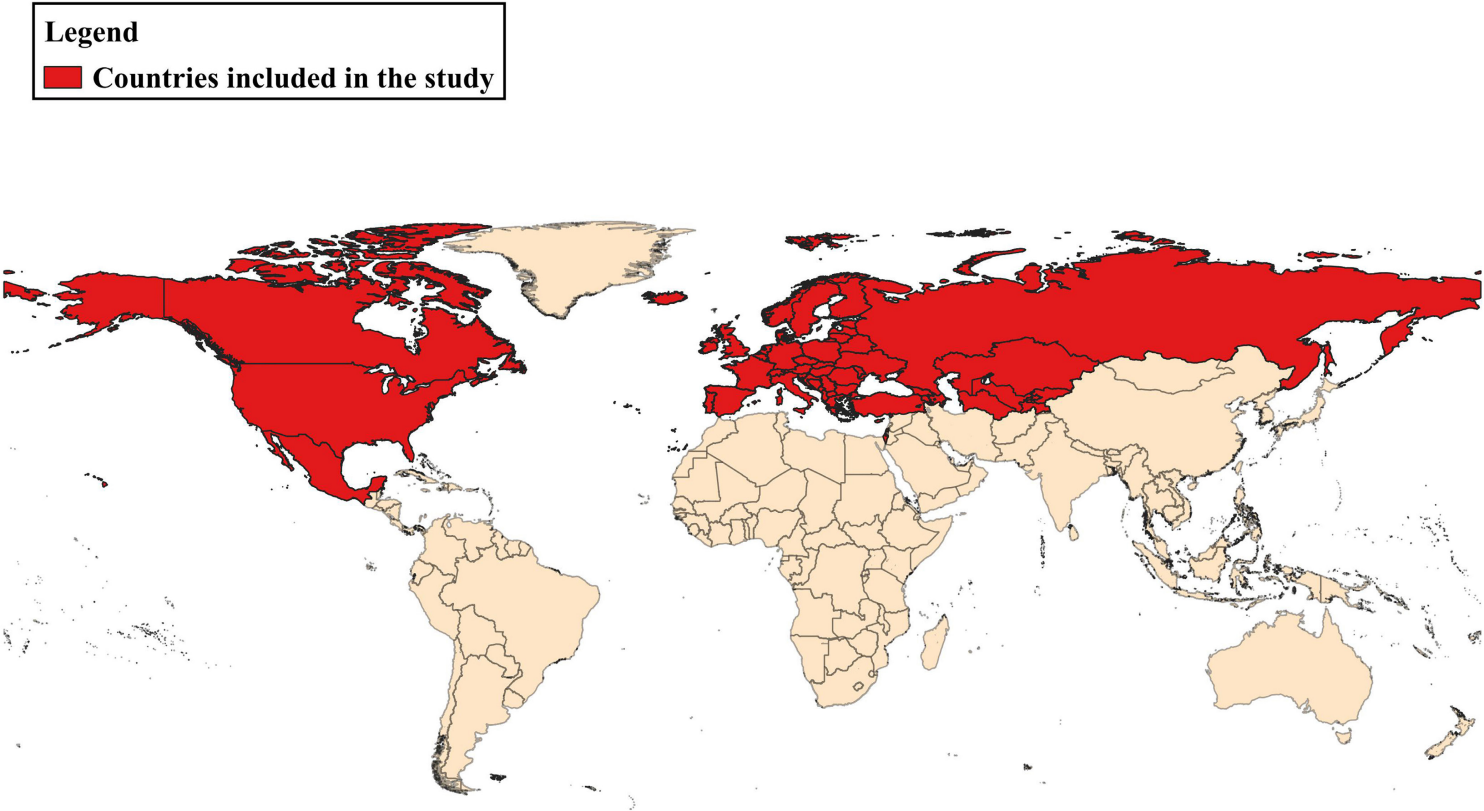
Countries included in the study

A ‘disease event’ is defined in this study as the reporting at country level of tularemia in lagomorphs (considered in this context as species included in the Leporidae family—hares and rabbits) in a specific year (during the period January 2014 to December 2019), including cases in humans linked to contact with lagomorphs. Lagomorphs are highly susceptible to the disease; hence, infection results in mortality (with high likelihood to be picked up by the media). As per the OIE definition, a tularemia event was considered only for infection caused by *Francisella tularensis*. This event definition was used to identify events in all the sources and homogenize the event detection for each source. A country was considered positive for tularemia occurrence, when at least one ‘tularemia event’ was recorded during one calendar year period. The geographic range considered included North America (United States, Canada and Mexico) and Europe (including the countries listed in that OIE Region)^5^ (Figure 1). Cases in humans not directly linked to contact with lagomorphs were excluded by the analysis. Disease events occurring in species other than lagomorphs were also excluded from the analysis.

The comparison among sources was done using the above definition of ‘event’. For the purpose of the CR method, only ‘TRUE’ events should be considered, in order to avoid an overestimation of the real number of events. While all the events reported in WAHIS are considered as reliable, being officially reported by National Veterinary Services, a deep screening of the events reported by the other unofficial sources was needed to remove unreliable reports.

### 2.3 Capture–recapture method

Capture–recapture models allow an estimation of the total number of events (detected and undetected by the sources) in a specific period and/or study area, for a specific disease. The sensitivity of the system is defined as the number of notified/detected events divided by the total number of events estimated by the CR model (German, 2000). While CR techniques have been widely applied to human diseases (Chao et al., 2001) and more recently to those affecting livestock (Vergne et al., 2015), they have never been employed for studying wildlife disease notification. As more than two data sources are explored, log‐linear models were implemented in this study. Using this approach, data are considered as a form of an incomplete 2*t* contingency table, where ‘*t*’ is the number of sources. The log‐linear method models the natural logarithm of the observed frequencies reported in cells of the contingency table as a linear combination of an intercept and source component terms. Direct and indirect dependences between data sources are handled using interaction terms (International Working Group for Disease Monitoring and Forecasting (IWGDMF), 1995).

### 2.4 Model implementation

The predicted number of tularemia events was firstly calculated using a three‐source CR log‐linear model, including the OIE‐WAHIS, EIOS and ProMED. Considering that scientific literature may provide information not reported by the other sources that gather closer to real‐time events, we built a four‐source CR log‐linear model which additionally included data retrieved from PubMed.

Homogeneity of time and place unit is an essential condition to avoid bias in event detection. Indeed, if the values provided by the sources are strongly linked to individual characteristics (e.g. year, region), a bias in the estimation of the total number of events is possible. So, it is important to select sources with homogeneous capture capacity and to carry out a stratification of the variables causing heterogeneity if needed. We evaluated the presence of potential heterogeneity among capture probabilities in the data set with exploratory heterogeneity graphs,^6^ while the dependence between data sources was modelled using interaction terms. Model selection was based on the Akaike information criterion (AIC), and the goodness‐of‐fit was assessed using the deviance statistic. Significance of main terms and interactions was set at 0.05. The 95% confidence intervals were computed assuming a Poisson distribution (Garwood, 1936). All analyses were carried out using the *Rcapture* package (Baillargeon & Rivest, 2007) in R software (R Core Team, 2018).

## 3 RESULTS

**FIGURE 2:**
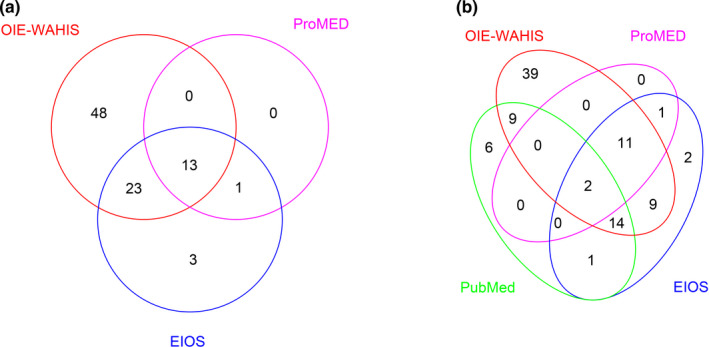
Illustration of the events identified by the three‐source (a), and four‐source (b) capture–recapture study

Schematic description of the distribution of the total number of cases from the three‐source and four‐source models, as well as their matching structure, is presented in Figure 2. There was a perfect matching of notifications between ProMED and EIOS, with a high dependency between the two sources. Based on the data identified within the sources, all events not notified (*n* = 10) to the OIE were from European countries, while all the events from North America were officially notified to the OIE. Specifically, 50% (5/10) of the unreported events occurred in Central Europe, 40% (4/10) in Eastern Europe and 10% (1/10) in Northern Europe.

For the three‐source approach, a total of 88 independent events were identified. The final log‐linear model (with smallest AIC) included all the individual effect terms and the interaction between ProMED and EIOS (Table 1). Except for ProMED and the interaction ProMED‐EIOS, all the terms were statistically significant (*p*‐value < .05). The predicted number of events was 93 (95%CI: 75–114; Table 1). Based on this, OIE‐WAHIS sensitivity was 90% (84/93). When accounting for records in scientific literature (PubMed source), the final log‐linear model (smallest AIC) contained the following interactions: OIE‐WAHIS*EIOS, ProMed*EIOS, ProMED*PubMed and EIOS*PubMed (Table 1). Also in this case, except for ProMED and the interaction ProMED‐EIOS, all the terms and interactions were statistically significant (*p*‐value < .05). Compared to the three‐source approach, the number of independent events increased to 94 and the estimate predicted by the four‐source model was 120 events (95% CI: 99–143). The sensitivity of the OIE‐WAHIS dropped in this case to 70% (84/120). As shown in the Table S2, a significant dependency was detected between most of the pairs of sources.

**TABLE 1:** Number of predicted events (N) modelling parameters from the application of the log‐linear model on three‐source and four‐source analyses

	*N* (se)	Deviance	*df*	AIC
Three‐source				
~ProMED*EIOS+OIE‐WAHIS	93.3 (3.7)	0.205	2	30.306
~OIE‐WAHIS*ProMED+ProMED*EIOS	94.3 (4.7)	0.000	1	32.101
~OIE‐WAHIS*EIOS+ProMED*EIOS	136.0 (2,867,831.0)°	0.205	1	32.306
~OIE‐WAHIS*ProMED+OIE‐WAHIS*EIOS+ProMED*EIOS	169.4 (8,017,478.9)°	0.000	0	34.101
~OIE‐WAHIS+ProMED+EIOS	91.7 (2.7)	27.014	3	55.114
~OIE‐WAHIS*EIOS+ProMED	88 (0.0)	25.525	2	55.626
~OIE‐WAHIS*ProMED+EIOS	91.9 (3.1)	26.975	2	57.076
~OIE‐WAHIS*ProMED+OIE‐WAHIS*EIOS	88.0 (0.0)	25.296	1	57.397
Four‐source				
~OIE‐WAHIS*EIOS+ProMed*EIOS+ProMED*PubMed+EIOS*PubMed	120.0 (15.2)	1.344	6	54.581
~OIE‐WAHIS*EIOS+OIE‐WAHIS*PubMed+ProMED*EIOS+ProMED*PubMed+EIOS*PubMed	156.4 (82.9)	0.755	5	55.993
~OIE‐WAHIS*ProMED+OIE‐WAHIS*EIOS+ProMED*EIOS+ProMED*PubMed+EIOS*PubMed	120.0 (15.2)	1.139	5	56.376
~ProMED*EIOS*PubMed+OIE‐WAHIS*EIOS	120.0 (15.2)	1.344	5	56.581
~ProMED*EIOS+ProMED*PubMed+EIOS*PubMed+OIE‐WAHIS	102.7 (4.4)	7.302	7	58.539
~OIE‐WAHIS*ProMED+ProMED*EIOS+ProMED*PubMed+EIOS*PubMed	105.0 (5.6)	5.507	6	58.745
~OIE‐WAHIS*EIOS+ProMED*EIOS+EIOS*PubMed	120.0 (15.2)	8.983	7	60.220
~OIE‐WAHIS*EIOS+ProMED*EIOS+PubMed	108.3 (7.7)	14.933	8	64.170
~ProMED*EIOS+EIOS*PubMed+OIE‐WAHIS	102.7 (4.4)	14.941	8	64.179

Note

The asterisk (*) is used to indicate all main effects and interactions. Please note that not all combinations are included.

°Warning indicating that the model fit is questionable occurred (algorithm did not converge, non‐positive sigma estimates for a normal heterogeneous model or large asymptotic bias).

## 4 DISCUSSION

The most important indicator of the efficiency and reliability of a surveillance system is represented by its capacity to detect the majority of the events being monitored—the higher the sensitivity of a surveillance system, the lower the number of undetected events. This is particularly relevant for international surveillance systems, like the OIE‐WAHIS, whose main aim is to reduce the transboundary spread of infectious diseases as much as possible. In this study, CR methods were used to estimate the number of tularemia events occurring during the period 2014 to 2019 in North America and Europe, and to evaluate the sensitivity of the OIE‐WAHIS system. The number of predicted events was 93 (95% CI: 75–114) and 120 (95% CI: 99–143), based on three‐source and four‐source log‐linear models, respectively. It appears clear from this study that the OIE‐WAHIS system performed efficiently for tularemia when considering a three‐source model and information coming from media reports, but it had a markedly lower sensitivity when accounting for events reported in the scientific literature. Even though the system sensitivity seems to be quite satisfactory, evidence of unreported events shows that the transparency of international reporting can still be further improved in specific areas, periods, and regions.

On the contrary, the three‐source model evidenced the sensitivity of an international reporting system related to sources with close to real‐time news publication, important for efficient disease monitoring. The information coming from scientific literature, having a lower dependency with the other sources and being consequently very useful to complement the global picture of a disease distribution, is more relevant for retrospective analysis that is generally beyond the scope of an efficient reporting system.

The CR approach to quantitatively evaluate the reporting of diseases and sensitivity of surveillance systems has already been applied in the field of veterinary epidemiology (Böhning et al., 2011; Del Rio Vilas et al., 2005; Pekova et al., 2017; Vergne, Calavas, et al., 2012; Vergne, Grosbois, et al., 2012). However, our study is the first to apply CR methods in the context of wildlife disease notification, and it is the first published study using data retrieved from the OIE‐WAHIS system. Admittedly, a master's thesis^7^ showed this first, obtaining similar results: sensitivity of 92% [IC 95% = 89–93] for the OIE‐WAHIS and 57% [IC 95% = 55–58] for PROMED for all OIE‐listed diseases events between 2005 and 2010.

Considering the important role of disease notification in wildlife, in light of recent pathogen spillovers with public health concern, quantification of the sensitivity of a surveillance system and the identification of key factors to improve the surveillance system are of pivotal importance to enhance early detection and early warning of emerging infectious diseases at national and international levels.

The OIE‐WAHIS is a unique system in the field of animal health. The information retrieved provides a global picture of the official animal disease situation as reported by National Veterinary Services. As it appears from this study, not all disease events occurring at the country level are reported to the OIE‐WAHIS, and discrepancies with the real‐life situation are possible. The quality and completeness of the OIE‐WAHIS data is influenced by the reporting behaviour of countries, by the efficiency and surveillance capacity of National Veterinary Services, and, in case of diseases occurring in wildlife, by an effective wildlife surveillance system in place at country level. To date, several studies have used the official information reported by National Veterinary Services to the OIE to understand the evolution of diseases at global and regional level (Cárdenas et al., 2019; Fanelli et al., 2020, 2021; Fanelli & Tizzani, 2020; Meske et al., 2021), highlighting on the one hand the usefulness and relevance of having an international reporting system that gathers data in a reference data set, and on the other, some limitations on reliability of information provided. The analysis of official data may be very useful to highlight gaps in international disease reporting, and thus in health policy planning by National Veterinary Services (Stärk & Häsler, 2015). In this sense, CR approaches may help to measure the level of underreporting and provide a more accurate picture of the real disease situation.

In the three‐source framework, the final model selected was the one including ProMED–EIOS interaction. Although the interaction was not statistically significant, a high degree of matching between these two sources was expected as EIOS already includes ProMED reports as a source in its daily scanning. However, we could not exclude the possibility that the EIOS algorithm may have missed some events reported by ProMED, and thus, we included both sources and retained this term even if it was not significant. It is important to consider that both EIOS and ProMED rely on searches in global media sources (e.g. news wires and websites). The main difference is that ProMED communications include not only open‐source data but also an additional network of experts to collect and analyse reliability of the information (Yu & Madoff, 2004). Therefore, events published in ProMED undergo a screening process and thus are much lower in number compared with those detected by EIOS, which employs language‐specific keywords and algorithms to extract relevant data without an a posteriori selection. It is interesting that the dependencies between the pairs of sources are quite high, as highlighted by the odds ratios (Table S2). The dependencies between the OIE‐WAHIS data and the other sources, in particular, can be explained by the fact that the OIE use media reports to detect unreported events and contact the Members in case of inconsistencies with official reports. Nevertheless, these findings should be interpreted considering the 95% confidence intervals due to the relatively moderate sample size.

Inclusion of PubMed in the four‐source model allowed identification of a larger number of tularemia events; in fact, PubMed showed the lowest level of dependencies with the other sources. This finding highlights the fact that information retrieved from published scientific articles may significantly contribute to monitoring disease events, adjusting for under‐ascertainment. Nevertheless, the use of scientific literature to monitor disease event information has some drawbacks as it can be quite time‐consuming. Indeed, to be able to adequately identify all relevant scientific references, investigators usually search multiple databases, which results in a considerable number of articles to screen (Bramer et al., 2017). Additionally, scientific research may occur over a long period of time before analysis and publication and thus does not have the same early warning value of the other data sources have. In this study, we considered events retrieved only from PubMed, imposing some keyword constraints to improve the search. This can be considered a limitation of this study as it could have biased the real number of tularemia events reported in the literature, and in turn the predicted estimate. The use of data from literature may also have an additional limitation, related to the time needed for a scientific study to go through the peer‐review process and publication, resulting in delayed accessibility of some information. Scientific studies could also use methods not usually implemented in routine surveillance (e.g. serology, bio‐molecular techniques such as PCR, or other advanced laboratory techniques), so they could detect ‘events’ that would not be picked up by the other three sources (e.g. no evident clinical signs and smaller distribution). Additionally, the number of scientific studies published depends on a country's investment in research activities and might create some geographical bias in the model estimations.

The three‐ source and four‐source models selected did not included any factors that could have influenced the capture homogeneity (e.g. country, year and region) due to the too limited number of events retrieved, and this could be seen as a limitation of our analysis. An additional limitation of our study was that it did not consider the full epidemiological characteristics and cycle of the disease. The definition of an event in our study was limited to the occurrence of tularemia in lagomorphs or human cases linked to contact with lagomorphs. The exclusion of water‐borne and mosquito‐borne tularemia outbreaks could have underestimated the real number of events, since *Francisella tularensis*, the agent of tularemia, occurs in the environment due to contamination by infected animals (Gürcan, 2014). This is shown by the growing number of articles published on human cases due to contaminated environments or through arthropod vectors (Hennebique et al., 2019). Even with a very accurate event definition and a preliminary cleaning activity of the events collected by the different sources, it is possible to include false events that can bias our results.

Finally, it would be interesting to further investigate whether the size of the event influences its reporting/publication bias. Specifically, mortality events with a high number of animal cases / mortality and /or a linked human case may be more likely to be investigated, reported and picked up by the media, while single animal mortality events may be investigated as part of a scientific study (e.g. reported via peer‐reviewed literature).

Although multiple bibliographic databases were not included, the benefits of incorporating scientific articles, as highlighted in this study, may be an invaluable component to provide a more accurate estimate of the predicted number of events, and thus of the sensitivity of the reporting system. This finding also highlights a gap in communication between academia and National Veterinary Services and the need for more systematic collaborations to enhance surveillance systems for notifiable diseases.

In the four‐source CR approach, the final model contains several interactions. The interaction terms provide information on the presence and strength of dependences between pairs of sources, without differentiating between direct and indirect dependence (Hook & Regal, 1995). Given the level of matching, the interactions reflect a positive dependency between the sources. Indeed, the presence of tularemia events in both media (screened by EIOS and ProMED) and scientific literature is not surprising given the zoonotic characteristic of the disease. Indeed, in recent years, the engagement and interest of the media in public health topics have increasingly grown, accelerating the dissemination of reports of disease occurrence (Brownstein et al., 2009). Therefore, the inclusion of both types of sources has been demonstrated to be essential to verify the accuracy of official data reported to the OIE. It should be considered that aside from the data reported by the Members, the OIE performs an active search for non‐official information and rumours disseminated by the media or reported in peer‐reviewed scientific journals (OIE, 2021a), and our results highlight and confirm the importance of using unofficial sources to improve system sensitivity. In particular, the OIE uses EIOS as a tool to collect signals/rumours and this may explain the dependencies between the two sources in the second model. Nevertheless, the outputs of active searching activity could be highly variable based on the epidemiologic characteristics of a disease, reporting behaviour of OIE Members, surveillance activity at National level, coverage provided by media reports and level of interest for the specific disease, as well as the presence of specific research funding provided to academia.

Tularemia has few implications for international trade (and is therefore unlikely to be under reported owing to sensitivities around trade), and the reduced geographical range of the disease to countries with advanced surveillance systems in wildlife may lead to an overestimation in the sensitivity of the OIE‐WAHIS. Therefore, we cannot assume that the system sensitivity is the same for other diseases, particularly for novel emerging diseases or for those diseases that have a great trade impact. For these reasons, it would be very interesting to apply the approach described in this case study to other selected diseases, to have a broader idea of the variability of the international reporting system, according to the epidemiological characteristic and impact of the diseases. The sensitivity of disease notification is generally higher for zoonotic diseases where the occurrence of human cases may serve as a sentinel for the detection of the disease in animals, due to the general better surveillance systems for public health and more apparent clinical presentation of some zoonoses in humans (compared to animals). On the contrary, the sensitivity of surveillance systems might be lower for more neglected diseases. Finally, the reporting behaviour of countries may also play a role in determining the system sensitivity; tularemia is in fact ‘endemic’ in a large number of the reporting countries and mostly reported through ‘six‐monthly reports’, with a possible difference in efficiency of National Veterinary Services in disease surveillance and detection. Considering this, it would be necessary to perform a much wider sensitivity analysis, including diseases with different epidemiological pattern, geographic range, and impacts on animal and public health. This will allow an assessment of priority unreported diseases, for which the sensitivity of international reporting system is lower and on which the actions of the Wildlife Health Framework should focus.

## 5 CONCLUSION

To our knowledge, this is the first application of CR methodology to assess the sensitivity of a notification system for wildlife diseases for a specific disease and regions. Overall, the sensitivity of the OIE‐WAHIS system for tularemia events was high, even if markedly reduced in the four‐source approach. This evaluation provides a positive evaluation of the role of international reporting for proper detection of disease events at the wildlife–human interface.

## CONFLICT OF INTEREST

The authors declare that there is no conflict of interest.

## AUTHOR CONTRIBUTIONS

Paolo Tizzani conceptualized the study, supervised the study and wrote—review & editing. Angela Fanelli investigated the study, contributed to methodology, curated the data, involved in formal analysis and wrote—original draft preparation. Lesa Thompson, Tiggy Grillo, Itlala Gizo and Christine Leon Rolez wrote—review & editing and validated the study. Lina Awada, Paula Caceres‐Soto, François Diaz, Tiggy Grillo, Itlala Gizo, Keith Hamilton, Christine Leon Rolez, Peter Melens, Roberta Morales, Lina Mur, Sophie Muset, Lorenz Nake, Lesa Thompson and Chadia Wannous validated the study.

## ETHICAL APPROVAL

This research does not contain any studies with animals performed by any of the authors.

## Supporting information

Table S1‐S2

## Data Availability

Data sharing not applicable to this article as no datasets were generated or analysed during the current study.

## Footnotes

^1^

https://wahis.oie.int/#/home

^2^

https://www.who.int/initiatives/eios

^3^

https://promedmail.org/promed‐posts/

^4^

https://pubmed.ncbi.nlm.nih.gov/advanced/

^5^
Member countries of the OIE Regional Commission for Europe: https://www.oie.int/fileadmin/Home/eng/About_us/docs/pdf/CR2018/2020_Commission_Europe_A.pdf

^6^
The plot.descriptive function in the *Rcapture* package was used to produce exploratory heterogeneity graphs.

^7^

http://thsese.vet–alfort.fr/telecharger.php?id=1654
