# Supplementary material for: Sensitivity of an international notification system for wildlife diseases: A case study using the OIE‐WAHIS data on tularemia
Source: Zoonoses Public Health. 2022 Jan 29;69(4):286–94. doi: 10.1111/zph.12916 (PMC9306881; doi:10.1111/zph.12916)
Supplement: Supplementary file 1 — Table S1‐S2 [file ZPH-69-286-s001.docx]

**Table S1:** PubMed's Advanced Search using database-appropriate syntax. Last search was done on 21 March 2021.

| Database | Strategy | No. of publications |
| --- | --- | --- |
| PubMed | (((((((rabbit[Title/Abstract]) OR (rabbits[Title/Abstract])) OR (hare[Title/Abstract])) OR (hares[Title/Abstract])) OR (lepus[Title/Abstract])) OR (Oryctolagus[Title/Abstract])) AND (tularensis[Title/Abstract])) OR (tularemia[Title/Abstract]) | **663** |

Dependence between sources was assessed by calculating the odds ratio (95% CI) between the two sources, as proposed by Wittes et al. (1968, 1974)

**Table S2:** Dependence between the pairs of sources (alpha level=0.05)

| Pairs | OR [95%CI] | p-value |
| --- | --- | --- |
| OIE-WAHIS-ProMED | 44 [6- 1894] | <0.001 |
| OIE-WAHIS-EIOS | 45[15-181] | <0.001 |
| OIE-WAHIS-PubMed | 14 [6-41] | <0.001 |
| ProMED-EIOS | Not applicable | Not applicable |
| ProMED-PubMed | 2[0.2-8] | 0.63 |
| EIOS-PubMed | 13 [6-33] | <0.001 |

**References:**

Wittes JT, Colton T, Sidel VW. Capture-recapture methods for assessing the completeness of case ascertainment when using multiple information sources. *J Chronic Dis*. 1974;27:25–36.

Wittes J, Sidel VW. A generalization of the simple capture-recapture model with applications to epidemiological research. *J Chronic Dis*. 1968;21:287–301.
